# Supplementary material for: Experimentally Induced Dieback Conditions Limit Phragmites australis Growth
Source: Microorganisms. 2023 Mar 2;11(3):639. doi: 10.3390/microorganisms11030639 (PMC10054352; doi:10.3390/microorganisms11030639)
Supplement: Supplementary file 1 [file microorganisms-11-00639-s001.zip › microorganisms-2119026-supplementary.pdf]

Supplementary materials

Experimentally induced dieback conditions limit *Phragmites australis* growth

Wesley A. Bickford [1,*], Danielle S. Snow [2], McKenzie K. H. Smith [3], Kathryn L. Kingsley [4], James F. White [4] and Kurt P. Kowalski [1]

[1] Great Lakes Science Center, U.S. Geological Survey, 1451 Green Road, Ann Arbor, MI 48105, USA;
wbickford@usgs.gov (W.A.B); kkowalski@usgs.gov (K.P.K.)
[2] Akima Systems Engineering, Herndon, VA 20171, USA; dboshers@umich.edu
[3] Department of Ecology and Evolutionary Biology, Tulane University, New Orleans, LA 70118, USA; msmith88@tulane.edu
[4] Department of Plant Biology, Rutgers University, New Brunswick, NJ 08901, USA; kathryn.l.kingsley@gmail.com (K.L.K.); jwhite3728@gmail.com (J.F.W.)
* Correspondence: wbickford@usgs.gov

Note: Any use of trade, firm, or product names is for descriptive purposes only and does not imply endorsement by the U.S. Government. All data used to support this study are available at [1].

Table S1: Plant biomass data from a preliminary study measuring biomass response to various short-chain fatty acid treatments. Biomass measures were from total (above- and belowground) dry weights from each mesocosm. All treatment groups contained eight replicate mesocosms. All acids were applied at the 0.05 M application rate. Significance at α = 0.05 was assigned following an ANOVA with post-hoc Tukey HSD adjustment. Full dataset can be found at [1].

Treatment	Mean Biomass (g)	Standard Error	Significance
Control	43.83	± 3.56	b
Calcium Acetate	15.30	± 2.31	c
Calcium Butyrate	2.05	± 0.43	a
Calcium Cocktail	1.40	± 0.30	a
Sodium Acetate	7.40	± 1.92	ac
Sodium Butyrate	6.32	± 1.75	ac
Sodium Cocktail	3.09	± 0.95	a
Novyrate	15.89	± 4.25	c
Tributyrin	1.24	± 0.20	a

Table S2: Quantitative Polymerase Chain Reaction (qPCR) reagents and conditions used to assess abundance of bacteria in soils.

Reagent volumes per reaction (µL)						Reaction conditions (40 cycles)			Quality control	
**Primers**	**ROX reference dye (500x dilution)**	**Bovine serum albumin (20 mg/mL)**	**Molecular biology grade water**	**Brilliant III Ultra-Fast SYBR Green qPCR Master Mix**	**Target DNA**	**Initial denaturation**	**Denaturation**	**Annealing & Extension**	**Average efficiency**	**Average R^2**
0.438 (338f/518r)	0.375	2.5	7.75	12.5	1	95°C 03:00	95°C 00:05	60°C 00:10	94.8 ± 3.4%	0.99

Table S3: Polymerase Chain Reaction (PCR) Conditions and Primer Sequences used for amplicon sequencing of bacterial communities**.**

Primer Name	Primer Sequence	Fused Primer Length†	PCR Mastermix	PCR Conditions	Reference
515f	5′- GTGCCAGCMGCCGCGGTAA-3′	63 bp	17 μL Accuprime pfx Supermix<br>1.0 μL 20 μM 515F<br>1.0 μL 20 μM 806R<br>1.0 μL Template DNA	Initial denaturation: 95℃ for 2 min, 30 cycles, denaturation: 95 ℃ for 20 s, annealing: 55℃ for 15 s, extension: 72℃ for 5 min (10 min final extension)	[2]
806r	5′- GGACTACHVGGGTWTCTAAT′3′	69 bp			

† Full fused primer included Illumina adapter (29-bp forward; 24-bp reverse), 8-bp unique barcode, 10-bp pad, 2-bp linker followed by the gene specific primer). Reverse adapter is used with forward primer, forward adapter with reverse.

microorganisms

MDPI

Table S4: Proportion of mesocosms with white vs. non-white and rigid vs non-rigid roots following SCFA treatments in a) low concentration *Phragmites* mesocosms, b) high concentration *Phragmites* mesocosms, and c) high concentration mesocosms of *Phragmites* or native species. *P.aus = Phragmites australis; S.acu = Schoenoplectus acutus; S.pec = Spartina pectinate.*

**Experiment 1: 0.01 M**								
	Control		Ca Butyrate		Ca Propionate		Cocktail	
	Monthly	Weekly	Monthly	Weekly	Monthly	Weekly	Monthly	Weekly
Non-White Roots	0	0	0	0	0	0.17	0	0.17
White Roots	1.0	1.0	1.0	1.0	1.0	0.83	1.0	0.83
Non-Rigid	0	0	0.17	0	0	0.17	0	0.17
Rigid	1.0	1.0	0.83	1.0	1.0	0.83	1.0	0.83
**Experiment 2: 0.05 M**								
	Control		Ca Butyrate		Ca Propionate		Cocktail	
	Monthly	Weekly	Monthly	Weekly	Monthly	Weekly	Monthly	Weekly
Non-White Roots	0	0	0	0.67	0.17	0.67	0.33	0.67
White Roots	1.0	1.0	1.0	0.33	0.83	0.33	0.67	0.33
Non-Rigid	0	0	0	0.17	0.17	0.67	0.17	0.4
Rigid	1.0	1.0	1.0	0.83	0.83	0.33	0.83	0.6

**Experiment 3: 0.05 M**												
	Control			Ca Butyrate			Ca Propionate			Cocktail		
	P.aus	*S.acu*	*S.pec*	*P.aus*	*S.acu*	*S.pec*	*P.aus*	*S.acu*	*S.pec*	*P.aus*	*S.acu*	*S.pec*
Non-White Roots	0	1.0	0.17	0.67	1.0	0.5	0.67	1.0	0.33	0.67	1.0	0.83
White Roots	1.0	0	0.83	0.33	0	0.5	0.33	0	0.67	0.33	0	0.17
Non-Rigid	0	0.17	0.17	0.17	0.5	0.33	0.67	0.33	0.17	0.4	0.33	0.17
Rigid	1.0	0.83	0.83	0.83	0.5	0.67	0.33	0.67	0.83	0.6	0.67	0.83

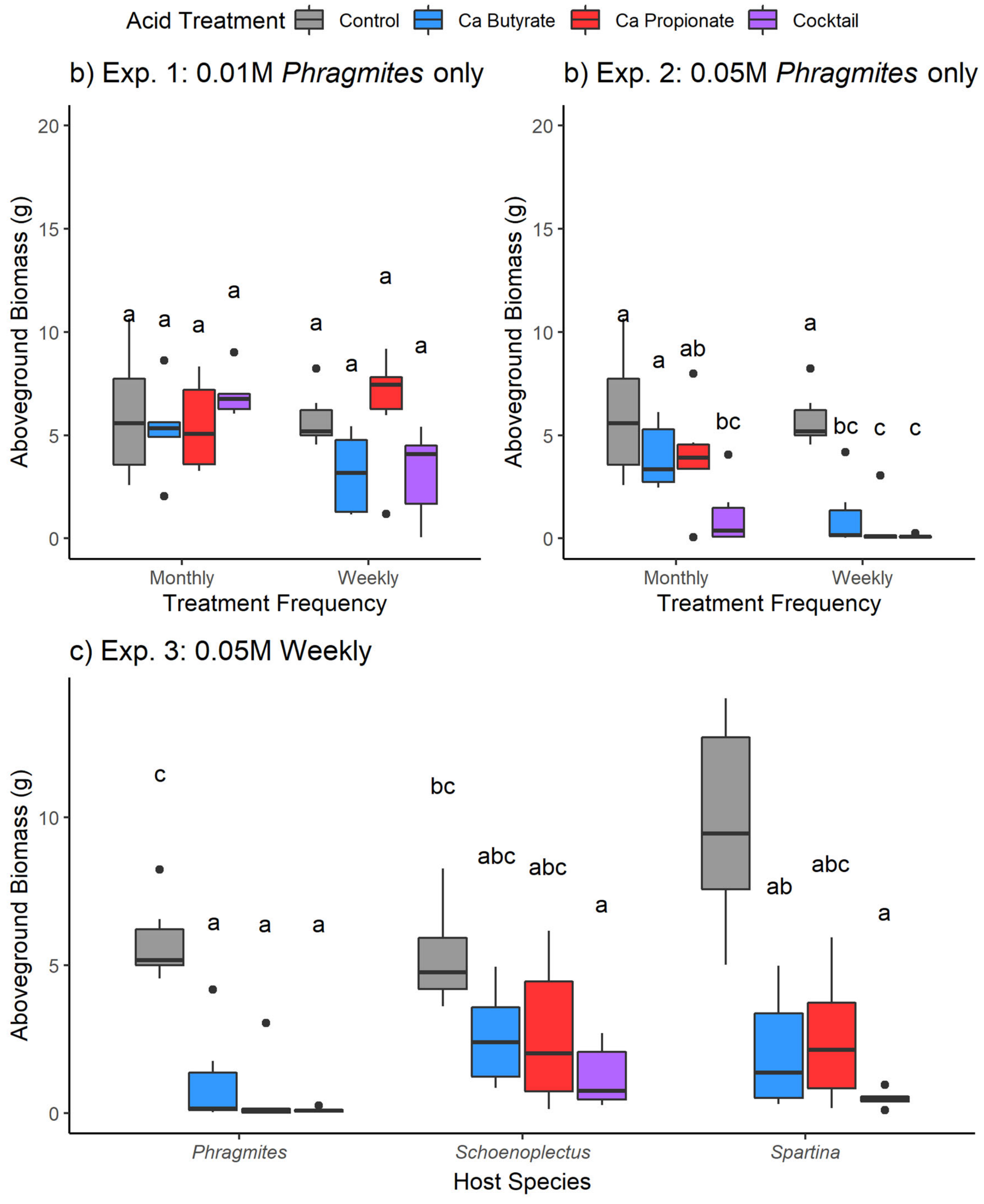

Figure S1: Aboveground plant biomass response to SCFA treatments in **a)** low concentration *Phragmites* mesocosms, **b)** high concentration *Phragmites* mesocosms, and **c)** high concentration mesocosms of *Phragmites* or native species. Colors indicate SCFA type. Letters indicate significant differences among all treatments within an experiment following ANOVA with post-hoc Tukey HSD adjustment.

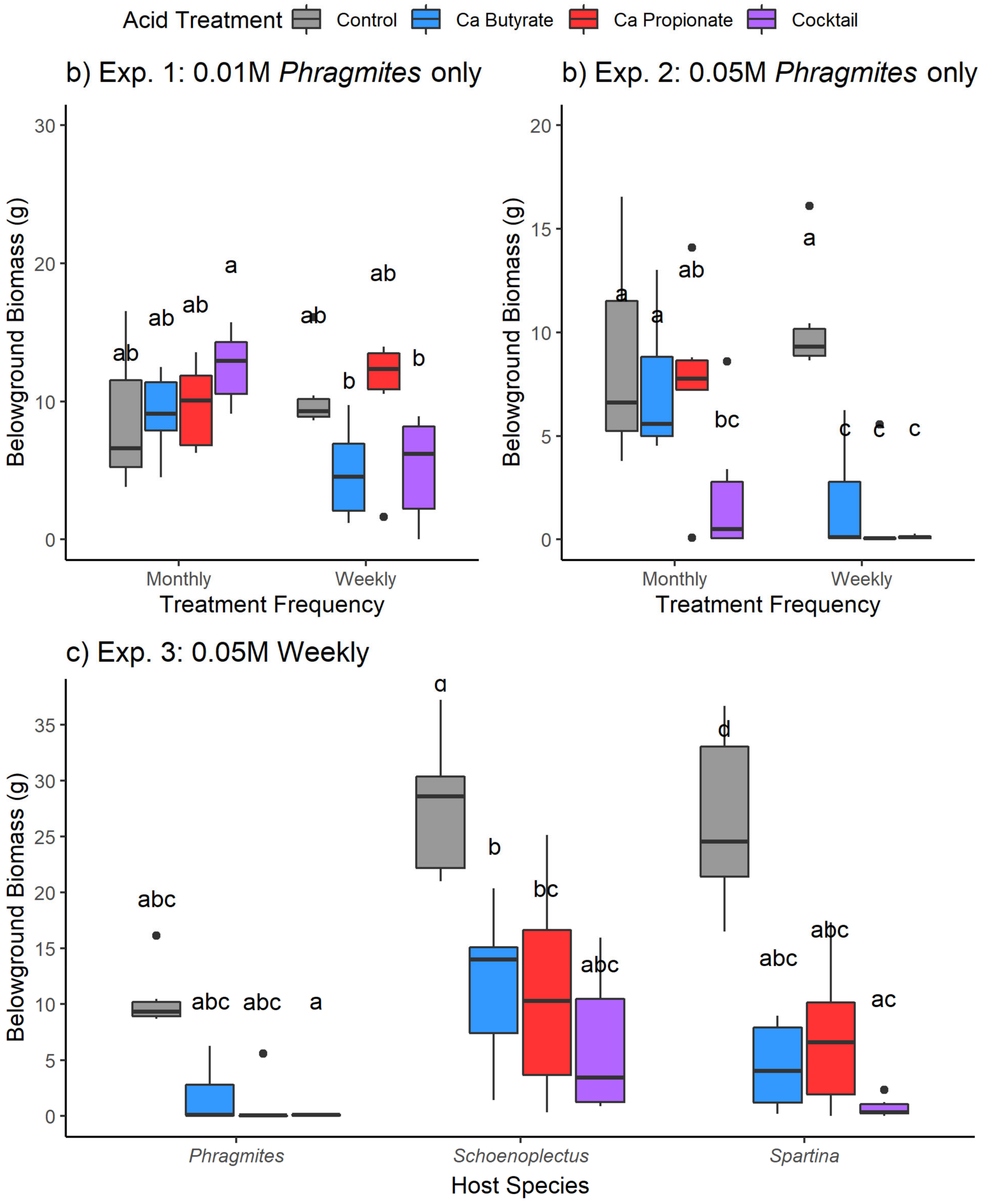

Figure S2: Belowground plant biomass response to SCFA treatments in **a)** low concentration *Phragmites* mesocosms, **b)** high concentration *Phragmites* mesocosms, and **c)** high concentration mesocosms of *Phragmites* or native species. Colors indicate SCFA type. Letters indicate significant differences among all treatments within an experiment following ANOVA with post-hoc Tukey HSD adjustment.

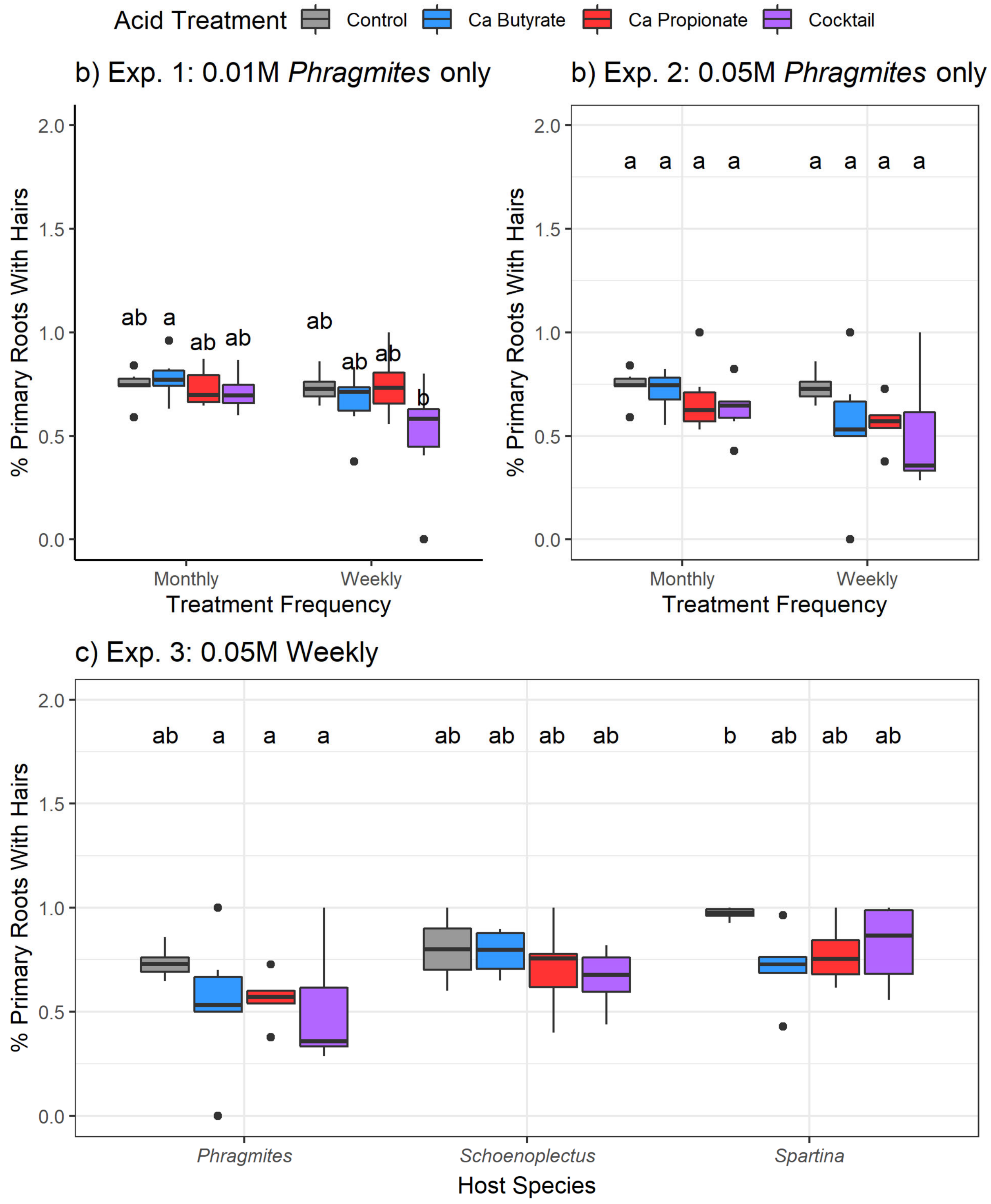

Figure S3: Root Hair response to SCFA treatments in **a)** low concentration *Phragmites* mesocosms, **b)** high concentration *Phragmites* mesocosms, and **c)** high concentration mesocosms of *Phragmites* or native species. Colors indicate SCFA type. Letters indicate significant differences among all treatments within an experiment following ANOVA with post-hoc Tukey HSD adjustment.

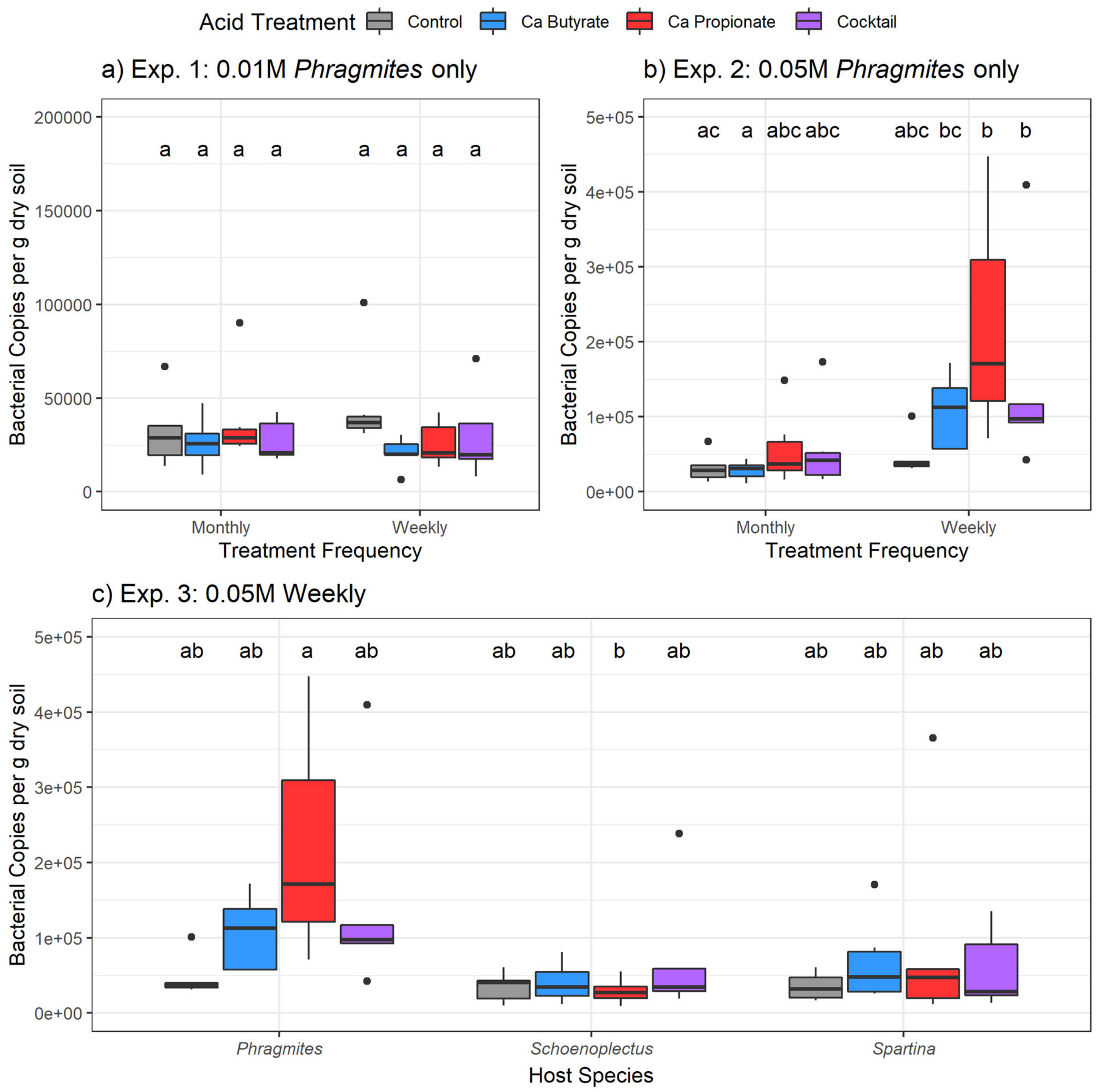

Figure S4: Rhizosphere bacterial abundance in response to SCFA treatments in **a)** low concentration *Phragmites* mesocosms, **b)** high concentration *Phragmites* mesocosms, and *c)* high concentration mesocosms of *Phragmites* or native species. Colors indicate SCFA type. Letters indicate significant differences among all treatments within an experiment following ANOVA with post-hoc Tukey HSD adjustment.

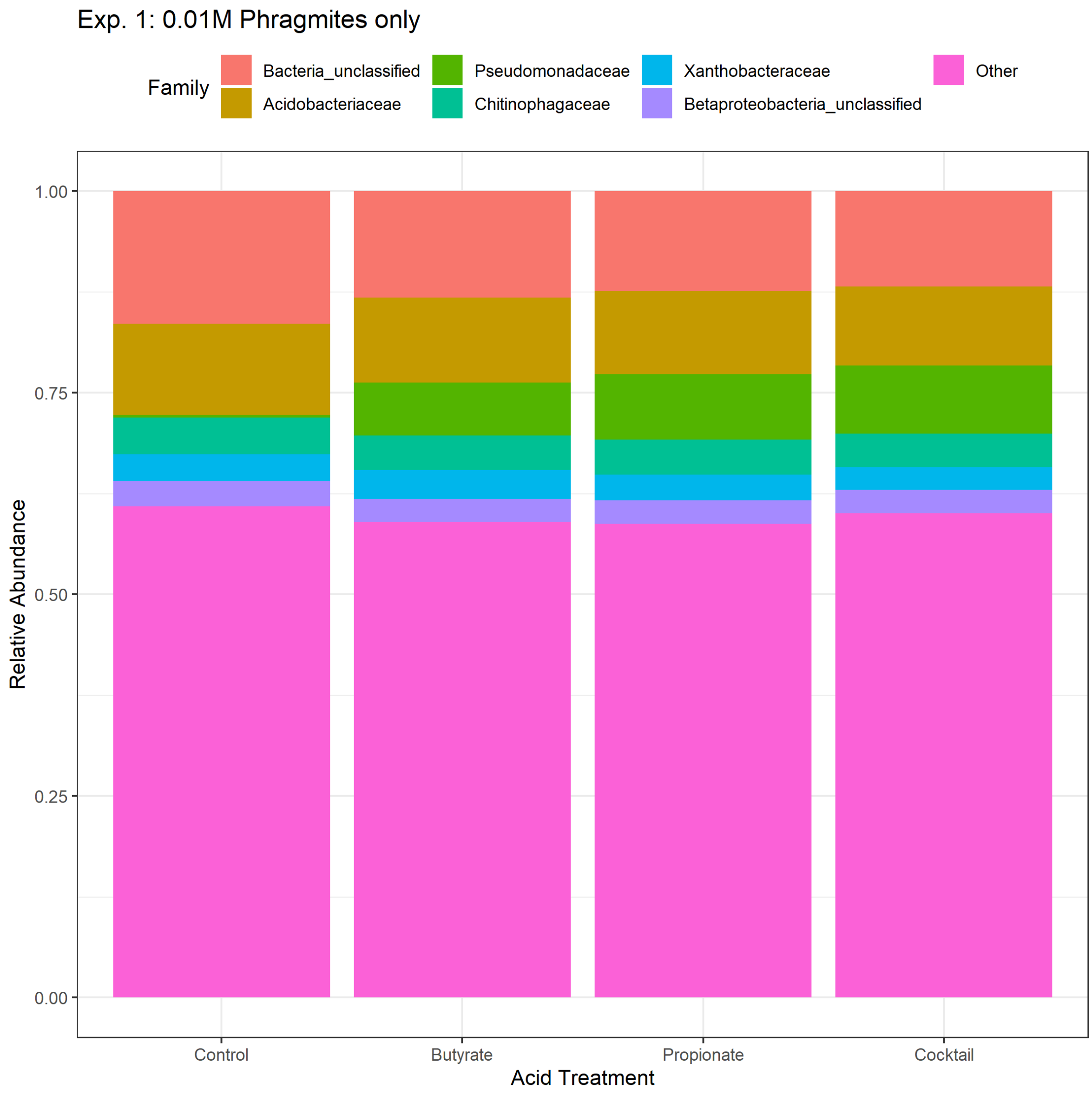

Figure S5: Relative abundance of bacterial families midway through Experiment 1 in response to each SCFA treatment. Colors indicate bacterial families. Only the top six families by relative abundance are represented. The “Other” category encompasses minor families outside of the top six.

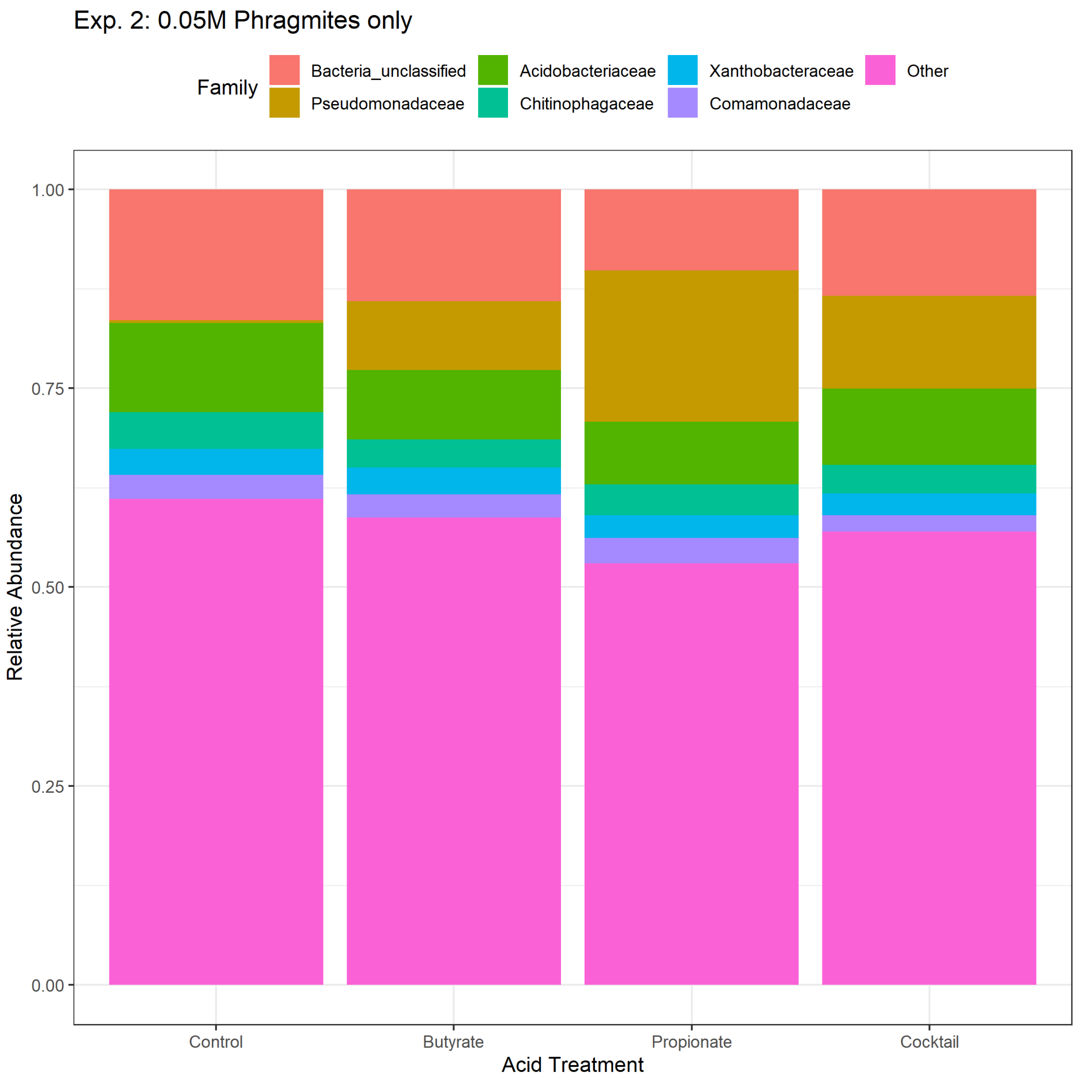

Figure S6: Relative abundance of bacterial families midway through Experiment 2 in response to each SCFA treatment. Colors indicate bacterial families. Only the top six families by relative abundance are represented. The “Other” category encompasses minor families outside of the top six.

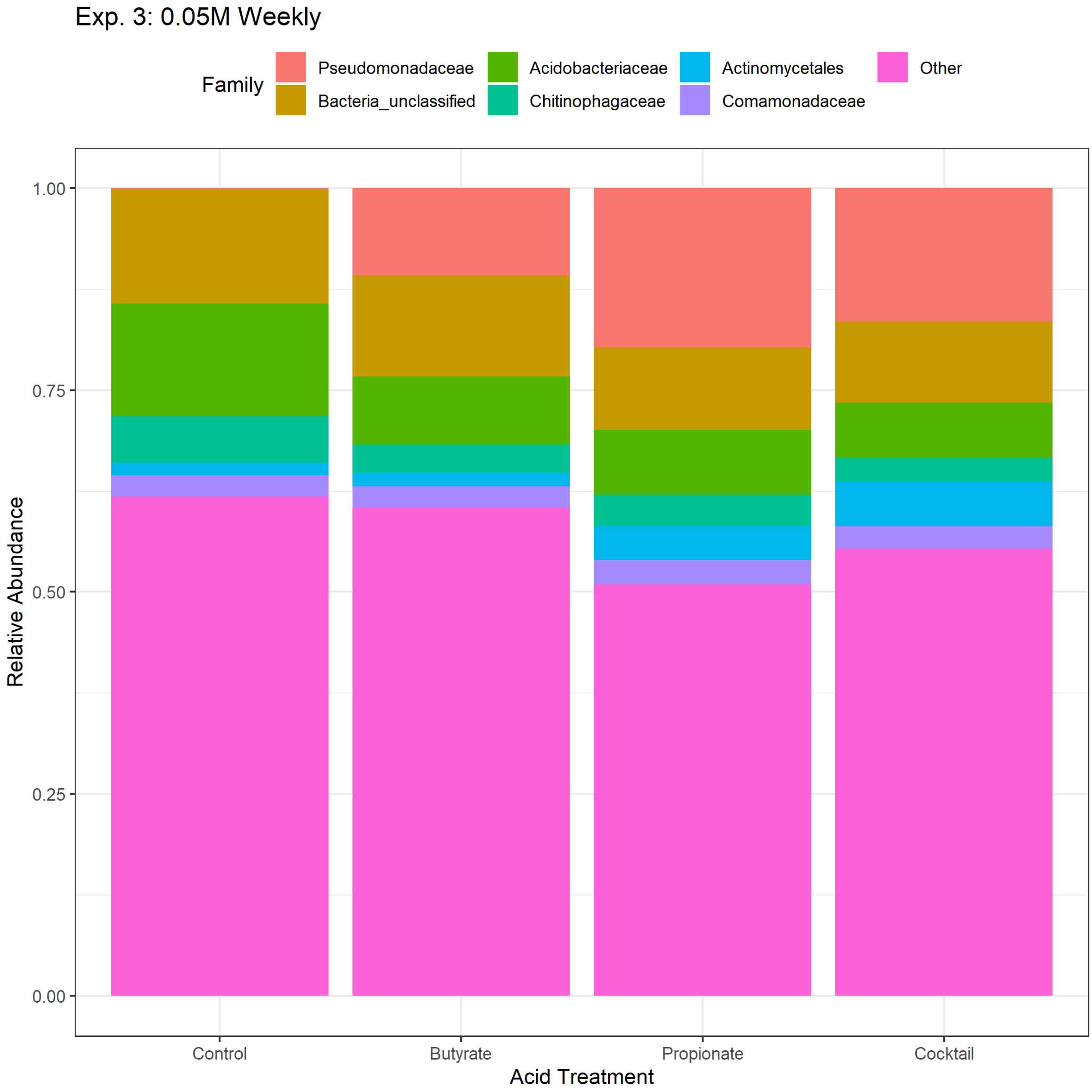

Figure S7: Relative abundance of bacterial families midway through Experiment 3 in response to each SCFA treatment. Colors indicate bacterial families. Only the top six families by relative abundance are represented. The "Other" category encompasses minor families outside of the top six.

References:

1. Bickford, W.A.; Snow, D.S.; Smith, M.K.H.; Kingsley, K.I.; White, J.F.; Kowalski, K.P. Plant and Microbial Response to Organic Acid Applications (2018-19 Experiment) **2023.** U.S. Geological Survey Data Release.
2. Kozich, J.J.; Westcott, S.L.; Baxter, N.T.; Highlander, S.K.; Schloss, P.D. Development of a Dual-Index Sequencing Strategy and Curation Pipeline for Analyzing Amplicon Sequence Data on the MiSeq. **2013**, *79*, 5112–5120, doi:10.1128/AEM.01043-13.
